# Barriers and facilitators to oral healthcare support in gestational diabetes mellitus: An interview study with healthcare professionals

**DOI:** 10.1111/dme.70181

**Published:** 2025-12-07

**Authors:** Camilla Böhme Kristensen, Koula Asimakopoulou, Mark Ide, Angus Forbes

**Affiliations:** ^1^ Centre for Host‐Microbiome Interactions King's College London London UK; ^2^ Faculty of Health and Life Sciences Oxford Brookes University Oxford UK; ^3^ Division of Care in Long Term Conditions King's College London London UK

**Keywords:** care delivery, gestational diabetes mellitus, health care professionals, oral health care, qualitative research, theoretical domains framework

## Abstract

**Aim:**

Periodontitis is a chronic inflammatory oral disease characterised by the persistent activation of immune cells, which contributes to insulin resistance and, consequently, an increased risk of systemic diseases, including gestational diabetes mellitus (GDM). At the same time, the risk of periodontitis is higher in individuals with diabetes, with higher glycaemic levels being the primary contributing factor. Oral health review is also advised in diabetes care by the NICE guidelines, because of its impact on blood glucose management. However, oral health may be overlooked in GDM due to the volume of other interventions required. Furthermore, little is known about the barriers and facilitators to oral healthcare support among healthcare professionals (HCPs) working in GDM care.

To examine the barriers and facilitators to oral healthcare support among HCPs in GDM care.

**Methods:**

The interview guide was based on the Theoretical Domains Framework (TDF), and semi‐structured interviews were used for data collection. The sample consisted of UK‐based HCPs with different professional backgrounds.

**Results:**

Four barriers and three facilitators were identified. These are related to the following TDF domains: professional role and identity; beliefs about consequences; knowledge; skills; memory, attention, and decision processes; and environmental context and resources.

**Conclusions:**

Oral health was not prioritised in GDM care. Furthermore, limited knowledge, increasing demands and time constraints, and fear of health‐related information overload were barriers to oral healthcare support. Training and education to increase knowledge, perceived importance of oral health and the HCPs' role and responsibilities facilitated oral healthcare support.

**What's new?**

- Oral health status impacts blood glucose management in diabetes. Gestational diabetes mellitus (GDM) pregnancies are marked by a higher prevalence of periodontal disease. Periodontal disease is also a risk factor for developing GDM.
- Little is known about the factors that impact motivation for oral healthcare support among non‐dental healthcare professionals (HCPs) working in diabetes care.
- We modelled the barriers and facilitators to oral healthcare support among HCPs specialising in GDM.
- This study forms part of a wider aim of developing a theory and evidence‐based toolkit to assist HCPs with implementing oral healthcare support in GDM management.

## 1 INTRODUCTION

Periodontitis is a chronic inflammatory oral disease of the periodontium, and a significant factor in pregnancy, potentially impacting maternal and infant health outcomes.^1^ Likewise, periodontitis is a key aspect of diabetes due to its impact on blood glucose management. The relationship between oral health and periodontitis and diabetes is bidirectional, with hyperglycaemia also impacting oral health status.^2^, ^3^ This prompted the NICE guidelines in 2022 to recommend regular oral health reviews as part of type 1 and type 2 diabetes management.^4^, ^5^ Oral health review entails person education, the adoption of oral self management behaviours and dental review and assessment.^6^ Gestational diabetes mellitus (GDM) is characterised by increased blood glucose levels in pregnancy. If not well managed, it is associated with several adverse health outcomes for the mother and infant.^6^ Oral health is likewise an important aspect of GDM. Periodontitis is more common and more severe in women experiencing GDM pregnancies when compared to women with normoglycaemic pregnancies.^7^ The relationship between oral health and GDM is also bidirectional, considering the association between baseline periodontitis and the incidence of GDM.^8^ An inflammatory host response is hypothesised to be the mechanism between oral health and diabetes.^9^

Emerging evidence also suggests that the severity of hyperglycaemia, rather than the GDM diagnosis alone, may be more predictive of adverse periodontal outcomes. For example, studies have shown that higher levels of HbA1c and fasting glucose correlate with greater periodontal pocket depth, clinical attachment loss and bleeding on probing among pregnant women, indicating a dose–response relationship between glycaemic dysregulation and periodontal health.^8^, ^10^ This is consistent with broader diabetes literature, where chronic hyperglycaemia is known to impair neutrophil function, increase advanced glycation end products (AGEs) and exacerbate inflammatory responses within periodontal tissues.^11^

In the other direction, the degree of periodontal inflammation reflected in the severity and extent of periodontitis or gingivitis may influence glycaemic control through systemic inflammatory pathways. Pro‐inflammatory cytokines such as IL‐6, TNF‐α and CRP, released from inflamed periodontal tissues, can exacerbate insulin resistance, potentially affecting glucose metabolism during pregnancy.^12^ While evidence in GDM populations remains somewhat limited compared to type 2 diabetes, studies such as that by Lira‐Junior et al.^13^ support a plausible biological mechanism linking periodontal inflammation with impaired glycaemic regulation in pregnancy.

Though oral health review forms part of standard diabetes NICE guidelines, it may be missed in GDM due to the number of other self management interventions required in this condition. Barriers to oral healthcare support among non‐dental healthcare professionals (HCPs) may also exacerbate this issue. Whilst the literature is limited, some evidence suggests that oral health is often overlooked in pregnancy care.^14^ Unawareness of oral health and poor referral pathways to dental services were preventing oral healthcare support among midwives.^15^ However, despite these barriers, prenatal care providers are positive towards oral health care, citing the importance of multidisciplinary approaches.^16^ In diabetes care, the barriers to oral healthcare support reported among General Practitioners were beliefs that oral health was outside their professional capacity. Other barriers included time constraints and a lack of referral pathways to dental services.^17^ While some of these barriers relate to structural issues in care delivery systems, it is nonetheless possible to address HCPs' awareness and beliefs to facilitate oral healthcare support.

To address oral healthcare support in diabetes, our group set out to develop a toolkit to assist non‐dental HCPs with providing oral health promotion in GDM management. First, we conducted an interview study with women with GDM to identify and model the determinants of oral healthcare behaviours.^6^ This study is the subsequent study in which we aimed to identify and model the barriers and facilitators to oral healthcare support among non‐dental HCPs specialising in GDM. Oral health support in this context entails providing basic information about oral health in diabetes, as directed by the NICE guidelines.^4^, ^5^ The evidence from both studies will be used to design the content in the proposed oral health toolkit.

## 2 METHODS

This study was an online cross‐sectional qualitative study. It was conducted to address the modelling process and outcomes phase in the Medical Research Council's framework for developing complex interventions. A favourable ethical opinion was obtained from King's College London's (KCL) research committee (reference: LRS/DP‐22/23–34,077). The study was reported according to the Consolidated Criteria for Reporting Qualitative Research Checklist.

### 2.1 Population and recruitment

UK‐based HCPs with experience in GDM care (e.g. diabetes specialist nurses, midwives, dieticians and consultants) were purposely sampled for the study. Prospect participants were eligible if they were currently or previously working with GDM in their HCP role, had access to a device with an Internet connection and spoke conversational English. The participants were recruited from Facebook groups such as Nurses United UK, the Diabetes Nurses UK Forum, Dieticians UK and Twitter using the study poster. The study was also advertised via the research institution's email bulletin. This is a biweekly service that advertises studies looking for participants. A £15 Amazon voucher was emailed to the participants after completion of the study.

### 2.2 Theoretical framework, data collection and procedure

To ensure a theoretical lens, the Theoretical Domains Framework (TDF) was used to develop the questions on the semi‐structured interview guide (Table 1). The TDF is a framework that proposes the different social, environmental, cognitive and affective influences that may prevent behaviour change. Each TDF domain links with specific behaviour change techniques, which help researchers with decision making in intervention design.^18^ It was initially developed to examine HCP behaviours but is also increasingly used in oral and dental research.^19^ The TDF is a well‐established theoretical framework that has been extensively used in qualitative research.^20^

**TABLE 1:** The TDF is a framework that proposes the different social, environmental, cognitive and affective influences that may prevent behaviour change (Cane et al.^18^). Its domains and definitions are described in this table.

Theoretical domain	Definition
Knowledge	An awareness of something
Skills	An ability or proficiency acquired through practice
Social/professional role and identity	A coherent set of behaviours and displayed personal qualities of an individual in a social or work setting
Belief about capabilities	Acceptance of the truth, reality or validity about an ability, talent or facility that a person can put to constructive use
Optimism	The confidence that things will happen for the best or that desired goals will be attained
Belief about consequences	Acceptance of the truth, reality or validity about outcomes of a behaviour in a given situation
Reinforcement	Increasing the probability of a response by arranging a dependent relationship, or contingency, between the response and a given stimulus
Intentions	A conscious decision to perform a behaviour or a resolve to act in a certain way
Goals	Mental representations of outcomes or end states that an individual wants to achieve
Memory, attention and decision processes	The ability to retain information, focus selectively on aspects of the environment and choose between two or more alternatives
Environmental context and resources	Any circumstance of a person's situation or environment that discourages or encourages the development of skills and abilities, independence, social competence and adaptive behaviour
Social influences	Those interpersonal processes that can cause individuals to change their thoughts, feelings or behaviours
Emotion	A complex reaction pattern, involving experiential, behavioural and physiological elements, by which the individual attempts to deal with a personally significant matter or event
Behavioural regulation	Anything aimed at managing or changing objectively observed or measured actions

All interviews were held and recorded on Microsoft Teams. Example questions on the interview guide included ‘Tell me your thoughts about oral health in GDM care?’ and ‘Describe what would enable/hinder you in oral health care support’. Key demographic information such as occupation, gender and age was also collected. Enrolled participants received the participant information sheet by email and signed the electronic consent form before completing the study. The principal researcher, a female PhD student (CBK), conducted the interviews in March 2023. Before the interview started, CBK provided a brief background about herself and the study objectives. Any questions from the participants were answered before commencing the interviews. CBK had received training in semi‐structured interview techniques from previous education and doctoral training courses. The interview guide was piloted with two PhD colleagues, and small revisions were made to some of the questions to enhance comprehension and clarity. The participants were at home or work when partaking in the interviews. CBK was alone in a meeting room in an office facility during all the interviews. The interviews lasted between 30 and 60 min. To incite in‐depth answers, probing following the DICE approach was used.^21^ Interview notes were taken by CBK immediately after the interviews.

### 2.3 Data analysis

A college‐approved third party transcribed the audio recordings (https://www.clearvoice.com/). The participants did not receive the transcripts for review or comments. The data management was facilitated by the NVivo software version 14. A two‐stage inductive and deductive approach, including a triangulation exercise, was used to analyse the data. The thematic analysis^22^ was carried out with the following five steps:

- Step 1: Familiarisation: CBK repeatedly read the transcribed interviews to increase familiarity with the text.
- Steps 2 and 3: Generation of initial codes and searching for themes: CBK coded the transcripts, and when saturation was reached, they were developed into initial themes. A colleague independent from the study reviewed two transcripts, codes and initial themes.
- Steps 4 and 5: Reviewing the themes and defining and naming the themes: The preliminary themes were revised, further developed and defined with KA and AF. Once the themes were finalised, they were deductively mapped onto the TDF.
- Definition of barriers and facilitators: An item within the data was identified as a barrier when it prevented or hindered the participants from engaging with oral health care support. A facilitator was identified as something that would favour, enable or help the participants with oral health care support.

## 3 RESULTS

During the call for participants, eight HCPs registered for the study, and all were enrolled and interviewed. No participants withdrew their interview data. Three participants had been introduced to CBK before the study. The sample consisted of three diabetes specialist nurses (one with a senior role); two diabetes specialist midwives (one with a lead role); a midwife, a nurse; and a diabetes dietician (Table 2).

**TABLE 2:** The characteristics of the study participants.

Characteristics	*N* and percentage
HCP role	
Diabetes specialist nurse	3 (38%)
Diabetes specialist midwife	2 (25%)
Midwife	1 (12%)
Nurse	1 (12%)
Diabetes dietician	1 (12%)
Age (years)	(*M* = 41.83 SD 10.19, range 28–51)^a^
Gender (female)	8 (100%)
Employed (yes)	8 (100%)
Ethnicity	
Caucasian	5 (63%)
Asian	1 (12.5%)
Other	1 (12.5%)
Mixed ethnicity	1 (12.5%)
Black	0
Civil status	
Married	5 (63%)
Living with a partner, not married	1 (12%)
Single	2 (25%)
Location	
Urban	6 (75%)
Rural	2 (25%)
Dental/oral health training	
No	7 (88%)
Yes	1 (12%)

^a^ Reported as mean, SD and minimum to maximum range.

### 3.1 Themes

Four barriers and three facilitators grouped under six TDF domains were identified. Table 3 contains the themes, illustrative quotes from various participants and the respective TDF domain.

**TABLE 3:** The themes (barriers and facilitators) derived from the study, supporting quotes from a range of participants and relevant TDF domains.

Themes	Illustrative quotes	TDF domain
Oral health agenda in GDM care (barrier)	A: ‘Oral health is not something that we discuss in our clinic’ (diabetes specialist nurse) B: ‘I've not heard anybody discuss oral health with their patients… I don't recall it being a topic’ (diabetes dietician) C: ‘Oral health is not something that we discuss on a regular basis… It is not discussed in team meetings or anything either’ (lead diabetes specialist midwife) D: ‘All pregnant women where I work are told that there is a higher chance of dental problems. And that's why they are entitled to free dental treatment during pregnancy… If anyone raises a dental issue, which they do occasionally, I'd always direct them to their dentist or advise them to get registered with one if they're not already. But that's about the limit of it really…’ (diabetes specialist midwife)	Professional role and identity
Limited oral health knowledge (barrier)	A: ‘The little oral health training I've had was part of my nursing training, and not in the context of diabetes’ (diabetes specialist nurse) B: ‘My knowledge base around oral health would be a barrier because I haven't had access to that information through training or courses’ (diabetes dietician) C: ‘I've done lots of different training programmes and oral health hasn't featured…For me, I would say it's about my own knowledge and my own appreciation for it’ (nurse)	Knowledge, skills
Increasing demands and time constraints (barrier)	A: ‘The gestational diabetes cohort is booming. There are more and more patients. The clinics are more and more oversubscribed. There's a lack of space to provide education. So, for instance, sometimes there is staff but there's not the actual physical location to take the patient in to deliver more in‐depth information’ (diabetes specialist nurse) B: ‘There are so many more women with GDM. I've been qualified for six years with three years of training before that, and in the nine years there are so many more people with GDM’ (midwife) C: ‘The numbers of GDM cases have grown over the years, and I think we are likely to see more ladies being diagnosed with GDM, particularly in my locality… So, we are capturing a lot more GDM ladies down our end’ (senior diabetes specialist nurse) D: ‘I think time is a definite issue’ (diabetes specialist nurse) E: ‘Probably also time constraints…The clinics are just inundated. So, I guess oral health is just an extra thing to talk about for HCPs. So, time is precious’ (midwife)	Environmental context and resources
Information overload (barrier)	A: ‘There's always the risk of information overload, particularly when people are initially diagnosed… They're learning this new way of life… And so, there is the potential for this overload of information’ (nurse) B: ‘You have to be a little bit careful with providing too much information because you can overwhelm the patients’ (lead diabetes specialist midwife) C: ‘And I think with pregnancy, there's also a bit of information overload…We have so much information that we need to support the ladies with’ (diabetes specialist nurse)	Memory, attention and decision processes
Oral health training and education (facilitator)	A: ‘I think education for me would be the big thing… If I had more information, I might be more inclined to be more active in promoting oral health’ (nurse) B: ‘I definitely would require some more support to have that understanding… If someone's talking about bleeding gums and where to signpost them, I definitely would need more training in that area’ (diabetes dietician) C: ‘There should be some training… If there is something worth us knowing about oral health’ (diabetes specialist midwife) D: ‘More training and a little bit more multidisciplinary work with specialists in the area of oral health, that would probably be the biggest facilitator’ (midwife)	Knowledge, skills
Role of non‐dental HCPs in oral health care delivery (facilitator)	A: ‘I would be quite happy to discuss oral health if I had training’ (diabetes dietician) B: ‘As a diabetes specialist nurse, we're completely, in the right place to provide oral health information and to provide education on oral health…’ (diabetes specialist nurse) C: ‘Everybody should know a little bit about oral health, so we can give advice. If our aim is to deliver a multi‐disciplinary team clinic, then everyone should know’ (diabetes specialist nurse) D: ‘I do think there is a role for nurses and for midwives to know more about oral health and to pass on those messages’ (nurse)	Professional role and identity
Perceived importance of oral health (facilitator)	A: ‘I think oral health is an important part of health and pregnancy’ (diabetes specialist nurse) B: ‘Oral health is really important in diabetes care broadly, and that would include GDM. I just think it's one of the areas in diabetes that hasn't got the attention it deserves because there's such a focus on other lifestyle modifications in GDM care’ (nurse) C: ‘I think oral health is important…. It's so intrinsically linked in diabetes’ (midwife)	Beliefs about consequences

### 3.2 Professional role and identity

#### 3.2.1 Barrier: Oral health agenda in GDM care

The impression was that oral health was not prioritised because the focus was on other GDM‐specific self management behaviours. This notion was shared among different types of HCPs. A diabetes specialist midwife further relayed how oral health was ‘not discussed in team meetings’, indicating that it is not prioritised on a wider team level either. Another diabetes specialist midwife shared how signposting to the dentist was occasionally part of her role. However, she acknowledged that the oral health discussions were limited to signposting, rather than providing oral self management instructions if a patient raised oral health concerns: ‘All pregnant women where I work are told that there is a higher chance of dental problems. And that's why they are entitled to free dental treatment during pregnancy… If anyone raises a dental issue, which they do occasionally, I'd always direct them to their dentist or advise them to get registered with one if they're not already. But that's about the limit of it really…’ (diabetes specialist midwife).

#### 3.2.2 Facilitator: Role of non‐dental HCPs in oral health care support

Many participants felt that their HCP role was an opportunity to discuss oral health care with their patients: ‘As a diabetes specialist nurse, we're completely in the right place to provide oral health information….’ Another participant relayed: ‘I do think there is a role for nurses and for midwives to know more about oral health and to pass on those messages’ (nurse). One participant furthermore described how oral healthcare support would facilitate multidisciplinary care for women with GDM: ‘If our aim is to deliver a multidisciplinary team clinic, then everyone should know (about oral health)…’ (diabetes specialist nurse).

### 3.3 Knowledge and skills

#### 3.3.1 Barrier: Limited oral health knowledge

Limited knowledge was a barrier to oral healthcare support, according to the participants. This finding was observed across HCP roles in the study group: ‘My knowledge base around oral health would be a barrier because I haven't had access to that information through training or courses’ (diabetes dietician); ‘I've done lots of different training programmes and oral health hasn't featured… (nurse). A diabetes specialist nurse further shared: “The little oral health training I've had was part of my nursing training, and not in the context of diabetes”’. The next theme illustrates the participants' views about oral health training and education.

#### 3.3.2 Facilitator: Oral health training and education

The participants believed that oral health training in the context of GDM was needed to facilitate oral healthcare support. This idea was also observed across different professions: ‘I think education for me would be the big thing… If I had more information, I might be more inclined to be more active in promoting oral health’ (nurse); ‘More training and a little bit more multidisciplinary work with specialists in the area of oral health, that would probably be the biggest facilitator’ (midwife). The diabetes dietician shared how more training would enable her to signpost the patients for help with their oral health: ‘I definitely would require some more support to have that understanding… If someone's talking about bleeding gums and where to signpost them, I definitely would need more training in that area’.

### 3.4 Environmental context and resources

#### 3.4.1 Barrier: Increasing demands and time constraints

Increasing demands due to the growing GDM prevalence negatively affected the care provided: ‘The gestational diabetes cohort is booming. There are more and more patients. The clinics are more and more oversubscribed. There's a lack of space to provide education. So, for instance, sometimes there is staff but there's not the actual physical location to take the patient in to deliver more in‐depth information’ (diabetes specialist nurse). The growing population of women with GDM also contributed to time constraints as noted by a midwife. She described how oral health would be an additional thing to discuss in an already busy consultation: ‘Probably also time constraints…The clinics are just inundated. So, I guess oral health is just an extra thing to talk about for HCPs. So, time is precious’ (midwife).

### 3.5 Memory, attention and decision processes

#### 3.5.1 Barrier: Information overload

Another barrier to oral healthcare support was the perceived risk of ‘information overload’, that is overwhelming the patients by sharing too much information with them.^17^ Given the volume of information already included in maternity and GDM care, the participants felt unsure whether adding oral health to the agenda would be problematic: ‘There's always the risk of information overload, particularly when people are initially diagnosed…’ (nurse); ‘And I think with pregnancy, there's also a bit of information overload… We have so much information that we need to support the ladies with’ (diabetes specialist nurse).

### 3.6 Beliefs about consequences

#### 3.6.1 Facilitator: Perceived importance of oral health in GDM

Despite the concerns with information overload, oral health care was considered an important part of health, pregnancy and diabetes among the participants: ‘I think oral health is an important part of health and pregnancy’ (diabetes specialist nurse); ‘I think oral health is important…. It's so intrinsically linked in diabetes’ (midwife). A nurse further contemplated how the other interventions required in GDM stole the focus from oral health care: ‘Oral health is really important in diabetes care broadly, and that would include GDM. I just think it's one of the areas in diabetes that hasn't got the attention it deserves because there's such a focus on lifestyle modifications in GDM care’.

## 4 DISCUSSION AND LOGIC MODEL

**FIGURE 1:**
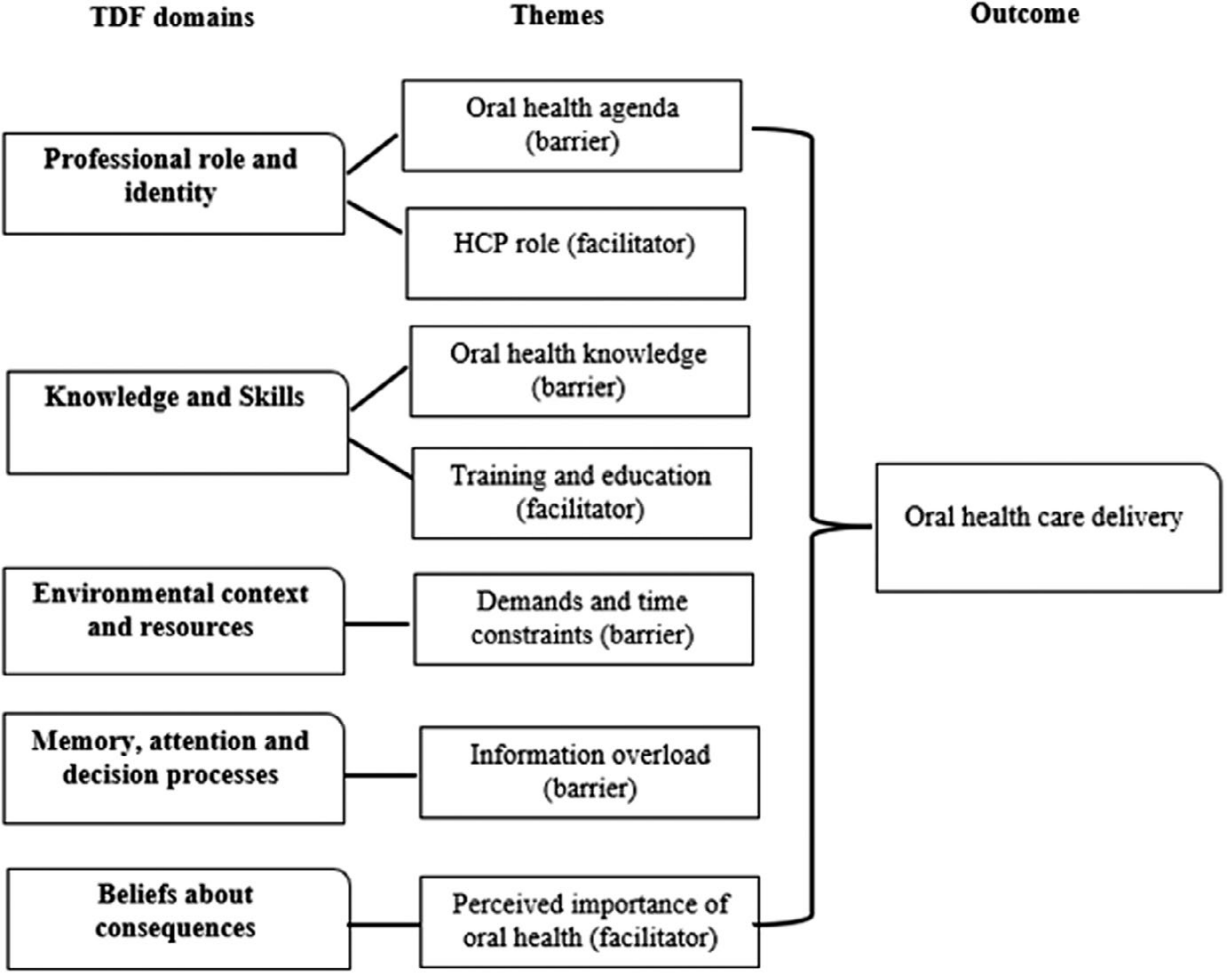
A logic model of the data collected, illustrating how the TDF domains, barriers and facilitators are theorised to impact oral health care delivery among non‐dental HCPs.

This study used the TDF to qualitatively explore the barriers and facilitators to oral healthcare support among HCPs specialising in GDM. Figure 1 illustrates the findings mapped into a logic model:

The ‘professional role and identity’ domain described the participants' views about their professional identity and responsibilities. Overall, oral health was not prioritised in GDM care, which is in line with previous research suggesting that it is often overlooked in diabetes and maternity care.^14^, ^15^, ^17^ Oversight of oral health has important implications for maternal and infant health outcomes. For example, oral health status impacts blood glucose management in diabetes,^2^ while higher glycaemic levels exacerbate the risks of diabetes‐related complications.^3^ Though the UK NICE guidelines recommend oral health review in maternity and diabetes care, it seems (at least based on this evidence) that there is a gap between recommendations and the practical implementation of such recommendations.^6^ This warrants the need for additional support and resources for HCPs in maternity and diabetes care who wish to ‘follow’ the NICE guidelines.

Up to 50% of pregnant women avoid the dentist even if they have dental issues.^15^ This is a concern, citing the potential impact of poor oral health on birth outcomes.^1^ The issue of non‐attendance persists in countries with free dental schemes for pregnant women (e.g. the UK).^15^ Our previous study indicated that long wait times and cancellations were among the factors that prevented women with GDM from attending the dentist.^6^ Other barriers include the costs of dental services.^15^ These obstacles to oral health care highlight the need for maternity HCPs to be involved in oral healthcare support, according to Vamos et al.^23^ An important facilitator in our current study was the view that oral health care was within the premises of the HCPs' roles. While one study found a positive inclination for adopting oral health review in maternity care,^14^ other research suggests that perinatal professionals are concerned about taking on this role.^17^ Regardless, including maternity HCPs in oral healthcare support may impact the high prevalence of non‐attendance among pregnant women. For example, discussing the importance of oral health care with pregnant women may prompt them to seek help. Furthermore, oral self management behaviours are critical in preventing periodontal disease.^24^ Discussing these in maternity and diabetes care may be an additional effective strategy in promoting oral health if patients are struggling to access the dentist. We do, however, acknowledge that optimal oral health care includes dental assessment and treatment (if needed) in addition to oral self management behaviours. Hence, the intention is not for non‐dental HCPs to provide dental treatment, but instead basic patient education and signposting.

The limited knowledge and lack of skills needed impacted the HCPs' motivation for oral healthcare support. This notion is supported by previous research among midwives.^15^ Similarly, we identified a lack of oral health awareness in our previous study with women with GDM.^6^ This suggests a need to address oral health knowledge among HCPs and patients alike to succeed with oral healthcare support in GDM.

Addressing antenatal professionals' oral health knowledge and skills; a controlled trial confirmed that the development and implementation of an oral health training programme was (i) effective in improving dental services uptake among pregnant women, and (ii) improved oral self care behaviours, the quality, awareness and knowledge of oral health.^25^ As illustrated by George et al.^25^ further training to increase HCPs' knowledge and skills can be achieved. In our present study, the HCPs reported that receiving further training and education would positively impact their motivation for addressing oral health care in GDM. Therefore, policymakers may need to revise the training provided for HCPs in GDM care to facilitate the knowledge and skills needed to take on oral health.

Increasing demands and time constraints were a barrier to oral healthcare support, citing the growing population of GDM pregnancies. Previous studies also highlight the lack of capacity to promote physical activity in GDM because of time constraints.^26^ This finding suggests that increasing demands and time constraints are issues pertinent to other GDM‐specific self care behaviours and do not exclusively apply to oral health. Lack of time during consultations impacts the patients' willingness to request further information about GDM, thereby decreasing the opportunity for patient‐centred care.^27^ Patient‐centred care entails a collaboration between HCPs and patients with a focus on care that accommodates the patients' biopsychosocial needs and preferences. It is associated with benefits for the patients and HCPs alike, including better health status, patient satisfaction, reduced use of care and greater work satisfaction.^28^ Considering the impact of the demands and time constraints on the care provided, there is a need to reconsider (i) strategies to address the mounting prevalence of diabetes, and (ii) the resources allocated to the healthcare system to manage these demands.

The barrier ‘information overload’ was grouped under the memory, attention and decision processes domain, because it describes the challenges with the HCPs' daily decision making. The impression was that the participants had to selectively choose what information they shared with their patients to prevent overwhelming them. The notion of information overload is supported by previous research indicating that women with GDM receive a lot of information and that HCPs are reluctant to overload their patients with additional information.^26^ Whilst providing health information is an important part of disease management, information overload may result in avoidance behaviour and non‐adherence to recommended self management behaviours.^29^ Though information overload is a real concern, our previous study demonstrated that women with GDM were highly motivated to engage in several self management behaviours to secure the health of their babies and themselves.^6^

Beliefs about the value of health behaviour have previously been found to impact HCPs' willingness to promote the health behaviour in question to women with GDM.^26^ This notion was also observed in our current study, with the participants expressing the belief that oral health was important. However, the view that oral health is important is interesting given that the HCPs in our study reported a general lack of oral health knowledge. Furthermore, the contrasting views between the perceived importance of oral health (facilitator) and the barrier of information overload were a noteworthy observation. Whilst the HCPs felt that oral health was important, they also believed that ‘adding’ more health information could overwhelm the patients. Another study may therefore be needed to examine non‐dental HCPs' ‘true feelings’ about oral healthcare support in GDM.

## 5 STRENGTHS AND LIMITATIONS

This study provides the starting point for initiating oral health care in diabetes by addressing the needs of the HCPs. However, the following study limitations should be noted. First, the study sample was rather small due to constraints on ethics and recruitment strategy. The sponsor's research and development team informed the researchers that even if NHS ethics were sought and approved, the principal researcher (CBK) could not attend secondary care settings to recruit participants because she is not a HCP. This hurdle, in addition to the lengthy process of NHS ethical clearance, resulted in the decision to obtain institutional clearance only and recruit participants from non‐clinical settings. The small sample in this study may impact the generalisability of the findings, and future research will need to include a wider range of HCPs and the male gender to increase diversity. Furthermore, solely relying on online recruitment may be associated with bias in the sample. Regardless, we attempted to recruit various HCPs involved in GDM care to ensure that a range of experiences were heard. To enhance the validity of the themes, we also created the thematic saturation table to illustrate the themes across the interviews. To support our sample size, we argue that previous qualitative studies have been published with samples under 10 according to a review by Marshall et al.^30^ Likewise, three to 20 participants have been proposed as an ideal sample size in descriptive qualitative studies.^31^ Another limitation of this study is the incentives used to attract participants. We cannot deny that some participants only participated for monetary reasons. However, each interviewee provided in‐depth responses, giving the impression that they were invested in the study. Lastly, the use of the TDF may have caused the oversight of other relevant variables in oral healthcare support among non‐dental HCPs.^32^ However, we attempted to address this limitation of the TDF by first inductively analysing the data.

## 6 CONCLUSIONS AND IMPLICATIONS

In conclusion, our study found that oral health was not prioritised in GDM care. Furthermore, limited knowledge, increasing demands and time constraints, and fear of health‐related information overload were barriers to oral health care support. On the contrary, training and education to increase knowledge and skills, the perceived importance of oral health and the HCPs' role and responsibilities were found to enable oral healthcare support. Our study may impact in two ways. First, these findings provide valuable insight into how oral health care can succeed in GDM management and may be of value to policymakers and clinicians alike. Second, this study will be used by our research group to inform a theory and evidence‐based toolkit to help non‐dental HCPs with oral health care support in GDM.

## FUNDING INFORMATION

This study is part of a doctoral research project funded by the Faculty of Dentistry, Oral and Craniofacial Sciences at King's College London.

## CONFLICT OF INTEREST STATEMENT

The authors declare no conflicts of interest.

## Data Availability

The data that support the findings of this study are available from the corresponding author upon reasonable request.
